# TRIM26 Negatively Regulates Interferon-β Production and Antiviral Response through Polyubiquitination and Degradation of Nuclear IRF3

**DOI:** 10.1371/journal.ppat.1004726

**Published:** 2015-03-12

**Authors:** Peng Wang, Wei Zhao, Kai Zhao, Lei Zhang, Chengjiang Gao

**Affiliations:** Department of Immunology & Key Laboratory of Infection and Immunity of Shandong Province, the School of Medicine, Shandong University, Jinan, Shandong, China; Kyoto University, JAPAN

## Abstract

Virus infection leads to the activation of transcription factor IRF3 and subsequent production of type I inteferons, which induce the transcription of various antiviral genes called interferon stimulated genes (ISGs) to eliminate viral infection. IRF3 activation requires phosphorylation, dimerization and nuclear translocation. However, the mechanisms for the termination of IRF3 activation in nucleus are elusive. Here we report the identification of TRIM26 to negatively regulate IFN-β production and antiviral response by targeting nuclear IRF3. TRIM26 bound to IRF3 and promoted its K48-linked polyubiquitination and degradation in nucleus. TRIM26 degraded WT IRF3 and the constitutive active mutant IRF3 5D, but not the phosphorylation deficient mutant IRF3 5A. Furthermore, IRF3 mutant in the Nuclear Localization Signal (NLS), which could not move into nucleus, was not degraded by TRIM26. Importantly, virus infection promoted TRIM26 nuclear translocation, which was required for IRF3 degradation. As a consequence, TRIM26 attenuated IFN-β promoter activation and IFN-β production downstream of TLR3/4, RLR and DNA sensing pathways. TRIM26 transgenic mice showed much less IRF3 activation and IFN-β production, while increased virus replication. Our findings delineate a novel mechanism for the termination of IRF3 activation in nucleus through TRIM26-mediated IRF3 ubiquitination and degradation.

## Author Summary

Innate immunity is the first line of defense to protect host from infection of invading pathogens. Production of type I inteferons by the innate immune cells is pivotal for the cellular antiviral immune responses. After virus infection, IFN-β transcription requires IRF3, which is activated through phosphorylation, dimerization and nuclear translocation. Although IRF3 activation and IFN-β production are essential for the host to prevent viral infection, aberrant or excessive IFN-β production may lead to the pathogenesis of human autoimmune diseases. Therefore, IRF3 activation and IFN-β production must be terminated at the appropriate time points after viral infection. Degradation of IRF3 in the nucleus represents a novel mechanism to terminate IFN-β production. Here we identified TRIM26 as a novel E3 ligase to target nuclear IRF3. TRIM26 attenuated IFN-β production through polyubiquitination and degradation of nuclear IRF3. *In vivo* experiments with TRIM26 transgenic mice further confirmed the negative function of TRIM26 on IFN-β production and antiviral responses. Given that IRF3 is a common molecule downstream of TLR3, RLRs and intracellular DNA signaling pathways, our study identified a novel mechanism to limit RNA and DNA virus-induced signaling, inflammation and tissue injury and provided new clues for the treatment of autoimmune diseases.

## Introduction

Innate immunity is essential for the host to protect from infection of invading pathogens. Activation of the innate immune response depends on the detection and recognition of pathogen-associated molecular patterns (PAMPs) by germline DNA-encoded pattern-recognition receptors (PRRs). The well studied PRRs include Toll-like receptors (TLRs), RIG-I-like receptors (RLRs), NOD-like receptors (NLRs) and intracellular DNA sensors [1], [2]. Among them, several types of PRR have been identified to recognize viral nucleic acid including RNA and DNA. For example, membrane-bound TLR3 recognizes extracellular viral double-stranded RNA in endosomes. Another type of RNA sensor is the cytosolic RLRs including RIG-I and MDA5, which detect intracellular viral dsRNA [3], [4]. Recently, several intracellular DNA sensors such as cGAS, IFI16, DDX41 and LRRFIP1 have been identified capable of sensing DNA from various microbes [5]-[8]. Upon binding with dsRNA, TLR3 triggers a signaling pathway mediated by Toll/IL-1R (TIR) domain-containing adaptor that induces IFN-β (TRIF) [9], [10]. While, RIG-I and MDA5 recruit a CARD-containing adapter protein MAVS (also known as VISA, IPS-1 and Cardif) to initiate the antiviral signaling pathway [11]-[14]. Although the nature of DNA sensors needs further investigation, the adaptor protein STING (also called MPYS, MITA, and ERIS) in DNA sensing pathway are well defined [15]-[17]. After viral infection, these key adaptors TRIF, MAVS and STING recruit the kinases TBK1 and IKKε to activate the transcription factor interferon-regulatory factor 3 (IRF3), leading to the production of type I inteferons and antiviral immune responses [18], [19]. Although IRF3 activation and IFN-β production are essential for the host to prevent viral infection, aberrant or excessive IFN-β production can lead to the pathogenesis of human autoimmune diseases such as SLE [20]. Therefore, IRF3 activation and IFN-β production must be terminated at the appropriate time points after viral infection.

IRF3 activation requires phosphorylation on multiple phosphorylation sites in the C-terminal of IRF3. After phosphorylation, IRF3 forms homo-dimmer and then moves into the nucleus, where it binds to target genes harboring interferon stimulation-response element (ISRE) [21]-[23]. Several mechanisms including dephosphorylation and polyubiquitination have been demonstrated to terminate IRF3 activation. Phosphorylated IRF3 was found to be dephosphorylated by phosphatase PP2A recruited by RACK1 adaptor protein. Therefore, RACK1 and PP2A limit virus-induced type I interferon signaling [24]. Phosphorylation in the C-terminal phosphor-acceptor has been reported to facilitate IRF3 proteasomal degradation after infection with SeV [23], [25]. But, the identity of the E3 ligase responsible for nuclear IRF3 ubiquitination and degradation is not defined.

TRIM26 is a member of the tripartite motif (TRIM) protein family composed of more than 70 members in human [26]. TRIM proteins share a similar characteristic structure, which includes a RING (R) domain, one or two B-boxes (B), and a coiled coil (CC) domain in the N-terminal and a domain in the C-terminal with variable structures. Here, we identified a novel function for TRIM26 as an E3 ubiquitin ligase for nuclear IRF3. TRIM26 bound to and induced IRF3 polyubiquitination in nucleus after virus infection. TRIM26 promoted the degradation of WT IRF3 and the phosphorylation active mutant IRF3 5D, but not the phosphorylation deficient mutant IRF3 5A. Nuclear translocation of IRF3 and TRIM26 was required for IRF3 degradation. TRIM26 transgenic mice had decreased IRF3 activation, IFN-β production and antiviral immune response. Our findings demonstrated that TRIM26 is essential for the termination of nuclear IRF3 activation.

## Results

### TRIM26 negatively regulates IFN-β production and antiviral response

**Fig 1:**
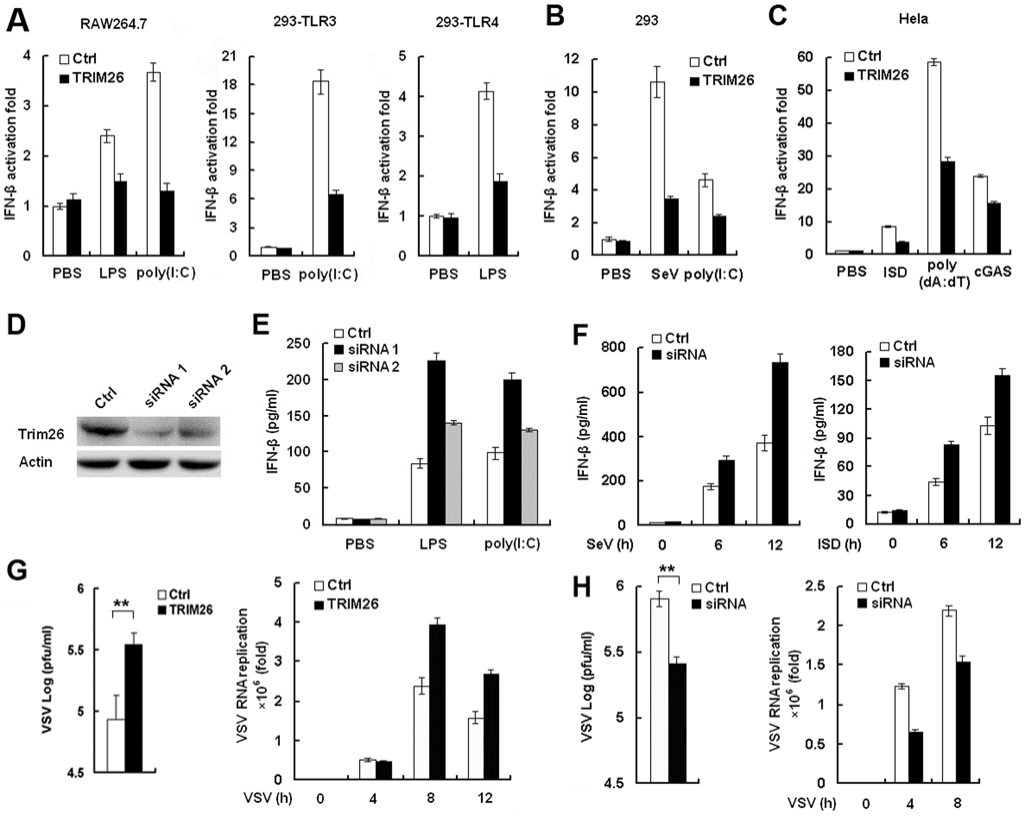
TRIM26 negatively regulates IFN-β production and antiviral response. (A) RAW264.7, HEK293/TLR4 and HEK293/TLR3 cells were transfected with IFN-β reporter plasmid together with TRIM26 expression plasmid or control plasmid, analyzed luciferase activity after treatment with LPS and poly(I:C), respectively. (B) HEK293 cells were transfected with IFN-β reporter plasmid together with TRIM26 expression plasmid or control plasmid, analyzed luciferase activity after infection with SeV or transfection with poly(I:C). (C) Hela cells were transfected with IFN-β reporter plasmid together with TRIM26 expression plasmid or control plasmid, analyzed luciferase activity after transfection with ISD, poly(dA:dT) and cGAS expression plasmid. (D) Western blot analysis of TRIM26 expression in mouse peritoneal macrophages transfected with control siRNA, TRIM26 siRNA 1 or siRNA 2 for 36 h. (E–F) ELISA analysis of IFN-β production in mice peritoneal macrophages transfected with TRIM26 siRNA as in (D) followed stimulation with LPS, poly(I:C), SeV or ISD. (G) Hela cells (2×10^5^) were transfected with the TRIM26 expression plasmid or control plasmid and then infected with VSV (MOI, 0.1). Supernatants were analyzed for VSV titers with standard plaque assays. Intracellular VSV RNA replicates and IFN-β expression were measured by QRT-PCR. (H) Mouse primary peritoneal macrophages were transfected with control siRNA (Ctrl) or TRIM26 siRNA (siRNA) and then infected with VSV (MOI, 0.1). VSV titers, intracellular VSV RNA replicates and IFN-β expression were measured as in (G). Data are representative of three independent experiments (mean ± S.D. of quadruplicates in A-C and triplicates in E–H).

TRIM26 is located in the MHC class I region [27], but its biological functions in the immune response remain elusive. To explore the function of TRIM26 in antiviral immune responses, the effect of TRIM26 on the activation of IFN-β expression downstream of various PRRs was investigated using IFN-β promoter reporter. LPS- and poly(I:C)-induced IFN-β promoter activation was attenuated in RAW264.7 macrophages transfected with TRIM26 expression plasmid, compared to that transfected with control vector (Fig. 1A). Similarly, transfection of TRIM26 expression plasmid also decreased LPS- and poly(I:C)-induced IFN-β activation in HEK293 cells stably expressing TLR4 and TLR3, respectively (Fig. 1A). Recognition of RNA virus through RIG-I like receptors (RLRs) may lead to the expression of IFN-β. Transfection of TRIM26 expression plasmid decreased SeV-induced IFN-β promoter activation in HEK293 cells (Fig. 1B). poly(I:C) present in the cytosol has been shown to activate IFN-β production through RIG-I and MDA-5 in HEK293 cells [4]. Consistent with the SeV infection data, IFN-β promoter activation induced by poly(I:C) transfection was also decreased upon TRIM26 overexpression (Fig. 1B). Recognition of DNA molecule through intracellular DNA sensors may also lead to the expression of IFN-β. To investigate whether DNA-induced IFN-β expression was affected by TRIM26, ISD (interferon-stimulating DNA) and poly(dA:dT) were transfeceted into Hela cells. Overexpression of TRIM26 attenuated ISD- and poly(dA:dT)-induced IFN-β promoter activation (Fig. 1C). Recent studies indicated that recognition of intracellular DNA molecule through cyclic GMP-AMP synthase (cGAS) led to the expression of IFN-β [5]. Overexpression of TRIM26 also attenuated cGAS-induced IFN-β promoter activation (Fig. 1C). These reporter assays strongly suggested that TRIM26 acts on molecules that are shared by various nucleic acid-induced signaling pathways to negatively regulate IFN-β expression.

To directly investigate the inhibitory role of TRIM26 in IFN-β production, two TRIM26 specific siRNAs were designed and transfected into peritoneal primary macrophages. Western blot analysis showed that the expression of TRIM26 protein was decreased after transfection with TRIM26 specific siRNA 1 and 2 (Fig. 1D). Transfection of TRIM26 siRNAs enhanced LPS- and poly(I:C)-induced IFN-β production (Fig. 1E). Importantly, TRIM26 siRNA 1, which has a higher efficiency to knockdown TRIM26 protein expression, has a greater potential to increase LPS- and poly(I:C)-induced IFN-β production (Fig. 1E). Therefore, TRIM26 siRNA 1 was used in the following experiments. Similarly, SeV- and ISD-induced IFN-β production was also increased in TRIM26 siRNA-transfected primary peritoneal macrophages (Fig. 1F). These results further confirmed the above reporter data and demonstrated that TRIM26 negatively regulates IFN-β production downstream of various PRRs.

IFN-β plays critical roles in the innate immune responses against viral infection. To directly investigate the effect of TRIM26 on antiviral responses, vesicular stomatitis virus (VSV) was used. Transfection of TRIM26 expression plasmid into Hela cells attenuated VSV-induced IFN-β expression (S1A Fig.), while VSV RNA replicates in the cells were increased in TRIM26-transfected cells (Fig. 1G). Accordingly, plaque assays showed that overexpression of TRIM26 substantially increased viral replication compared to control vector-transfected cells (Fig. 1G). In contrast, transfection of TRIM26 siRNA into primary peritoneal macrophages increased VSV-induced IFN-β expression (S1B Fig.), while intracellular VSV RNA replicates was decreased (Fig. 1H). Plaque assays showed that transfection of TRIM26 siRNA significantly decreased VSV viral replication (Fig. 1H). Taken together, these data demonstrated that TRIM26 negatively regulates IFN-β production and antiviral immune responses.

### TRIM26 targets IRF3

**Fig 2:**
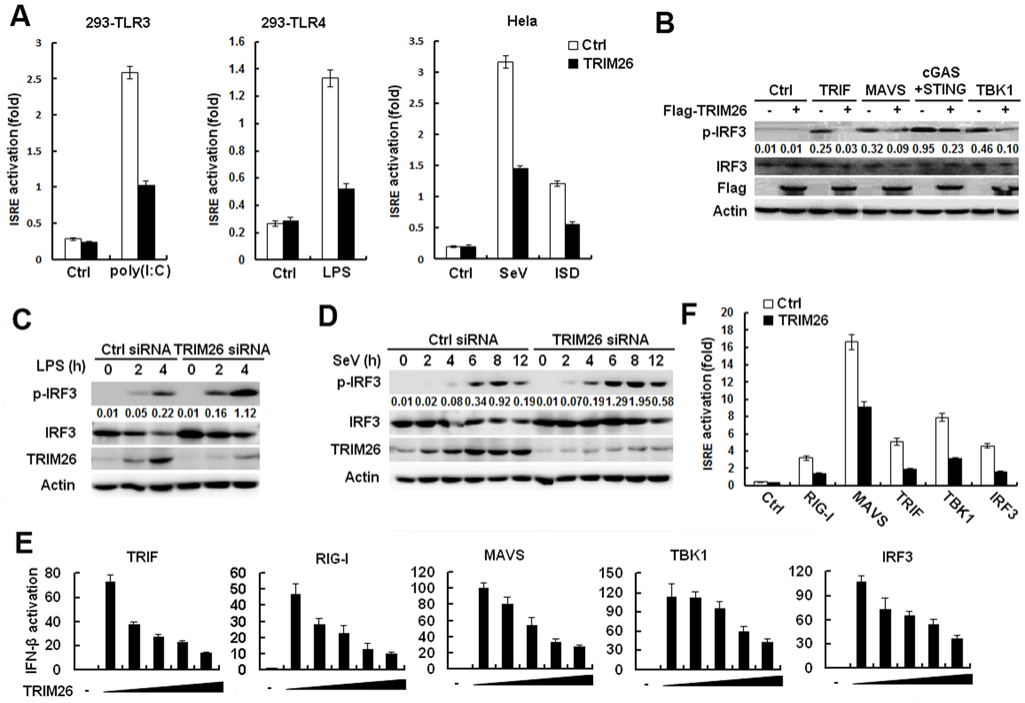
TRIM26 targets IRF3. (A) HEK293/TLR3 and HEK293/TLR4 or Hela cells were transfected with ISRE reporter plasmid together with TRIM26 expression plasmid or control plasmid, analyzed luciferase activity after treatment with indicated agonists. (B) Western blot analysis of phosphorylated-IRF3 and total IRF3 in HEK293 cells transfected with TRIM26 expression plasmid or control plasmid, along with indicated adaptors. (C-D) Western blot analysis of phosphorylated-IRF3, and total IRF3 in mouse peritoneal macrophages transfected with control siRNA (Ctrl) or TRIM26 siRNA 1 (siRNA) and stimulated with LPS (C) or infected with SeV (D) for indicated times. (E) HEK293 cells were transfected with expression plasmids for TRIF, RIG-I, MAVS, TBK1 or IRF3 5D, along with IFN-β reporter plasmid and increasing amount of TRIM26 plasmid, and analyzed luciferase activity. (F) HEK293 cells were transfected with expression plasmids for RIG-I, MAVS, TRIF, TBK1 or IRF3 5D, along with ISRE reporter plasmid and TRIM26 plasmid, and analyzed luciferase activity. Data are representative of at least three independent experiments (mean ± S.D. of quadruplicates in A, E, F).

IRF3 is the main transcription factor involved in IFN-β production. To investigate the effect of TRIM26 on IRF3 activation, several set of experiments were performed. LPS and poly(I:C) stimulation increased the activation of ISRE reporter in HEK293 cells stably expressing TLR4 and TLR3, respectively (Fig. 2A). While, transfection with TRIM26 expression plasmid attenuated LPS- and poly(I:C)-induced activation of ISRE reporter (Fig. 2A). Similarly, SeV and ISD-induced ISRE activation was also decreased by TRIM26 overexpression (Fig. 2A). Transfection of TRIF, MAVS, STING+cGAS and TBK1 could induce IRF3 phosphorylation in HEK293 cells (Fig. 2B). Overexpression of TRIM26 substantially decreased TRIF-, MAVS-, STING+cGAS- and TBK1-induced IRF3 phosphorylation (Fig. 2B). In contrast, knockdown of endogenous TRIM26 expression by siRNA in primary peritoneal macrophages increased LPS-induced IRF3 phosphorylation (Fig. 2C). Similarly, SeV-induced IRF3 phosphorylation was also increased in TRIM26 siRNA-transfected macrophages (Fig. 2D). These data suggested that TRIM26 inhibits IRF3 activation to negatively regulate IFN-β production.

To determine the molecular targets of TRIM26, the effect of TRIM26 overexpression on the activation of IFN-β promoter mediated by various molecules was examined in reporter assays. As shown in Fig. 2E, TRIF-, RIG-I-, MAVS-, TBK1- and IRF3-induced IFN-β promoter activation was inhibited by TRIM26 overexpression in a dose dependent manner. Similarly, TRIF-, RIG-I-, MAVS-, TBK1- and IRF3-induced activation of ISRE reporter was also inhibited by TRIM26 overexpression (Fig. 2F). These data indicated that TRIM26 targets IRF3 directly to regulate IFN-β production.

### TRIM26 promotes IRF3 ubiquitination and proteasomal degradation

**Fig 3:**
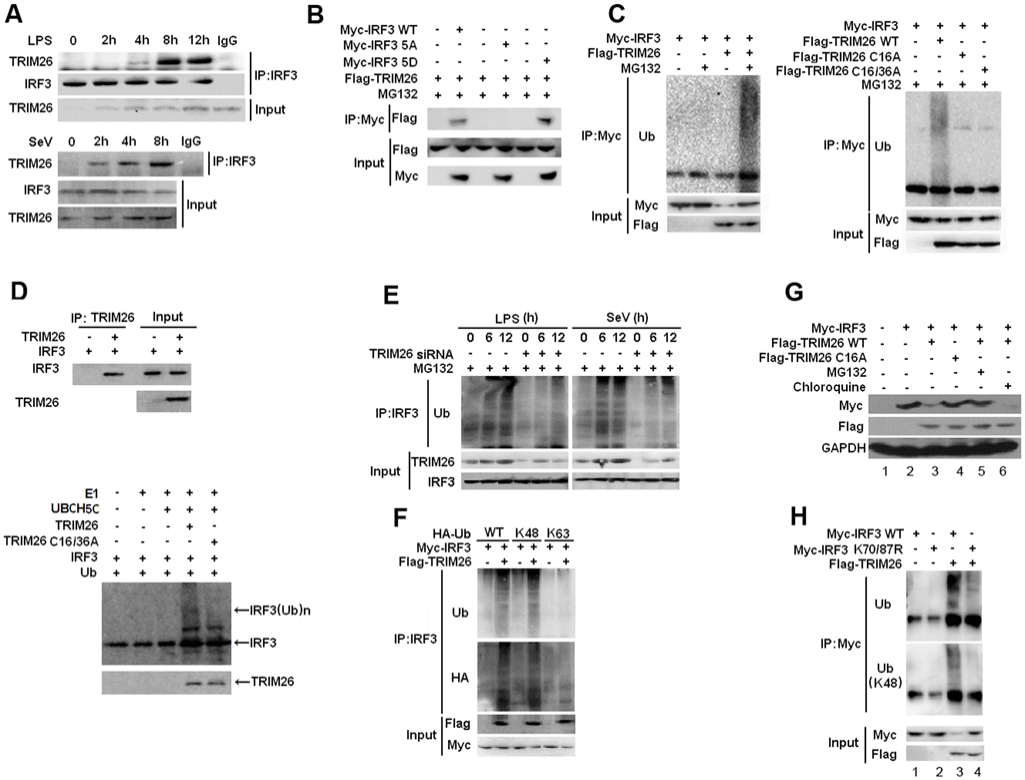
TRIM26 promotes IRF3 ubiquitination and proteasomal degradation. (A) Lysates from mice peritoneal macrophages stimulated with LPS or infected with SeV for indicated time periods were subjected to immunoprecipitation with anti-IRF3 antibody followed by western blot analysis with anti-TRIM26 antibody. (B) Lysates from HEK293 cells transfected with Flag-TRIM26 and IRF3 WT, 5D, 5A or control vector and treated with MG-132 were subjected to immunoprecipitation with anti-Myc antibody followed by western blot analysis with anti-Flag antibody. (C) Lysates from HEK293 cells transfected with expression plasmids for Myc-IRF3, Flag-TRIM26 WT, C16A, C16/36A and HA-Ub plasmids were subjected to immunoprecipitation with anti-Myc antibody followed by western blot analysis with anti-Ub antibody. (D) TRIM26 WT, C16/36A and WT IRF3 were obtained by *in vitro* transcription and translation. Interaction between TRIM26 and IRF3 was assayed by mixing WT TRIM26 and WT IRF3 together followed by IP with TRIM26 antibody and WB with IRF3 antibody. *In vitro* ubiquitination assay was performed in the presence of Ub, E1, UbcH5c, TRIM26 and IRF3. The ubiquitination of IRF3 was examined by WB with IRF3 antibody. (E) Lysates from mouse peritoneal macrophages transfected with control siRNA (Ctrl) or TRIM26 siRNA (siRNA) pretreated with MG-132 for 2 h followed with stimulation with LPS or infection with SeV for indicated time periods were subjected to immunoprecipitation with anti-IRF3 antibody followed by western blot analysis with anti-Ub antibody. (F) Lysates from HEK293 cells transiently transfected with expression plasmids for Myc-IRF3, Flag-TRIM26 and HA-Ub (WT), HA-Ub (K48) or HA-Ub (K63) were subjected to immunoprecipitation with anti-Myc antibody followed by western blot analysis with anti-Ub or anti-HA antibody. (G) Western blot analysis of Myc-IRF3 expression in HEK293 cells transfected with expression plasmids for Myc-IRF3, Flag-TRIM26 WT or TRIM26 C16A and then treated with MG132 or Chloroquine for 4 h. (H) Lysates from HEK293 cells transiently cotransfected with expression plasmids for Myc-IRF3 WT or K70/87R, along with Flag-TRIM26 and HA-Ub plasmids were subjected to immunoprecipitation with anti-Myc antibody followed by western blot analysis with anti-Ub or anti-Ub(K48) antibody. Similar results were obtained from three independent experiments.

The presence of RING-finger domain indicates TRIM26 may function as an E3 ligase. Thus, the ability of TRIM26 to induce IRF3 polyubiquitination and degradation was investigated. Co-Immunoprecipitation (Co-IP) showed that endogenous TRIM26 formed a complex with IRF3 upon LPS stimulation and SeV infection, while there is no interaction in untreated macrophages (Fig. 3A). Co-transfection of Flag-TRIM26 and Myc-IRF3 demonstrated that Flag-TRIM26 interacted with WT IRF3 and the constitutive active mutant IRF3 5D, but not with the non-active IRF3 mutant 5A (Fig. 3B).

To directly test TRIM26-mediated IRF3 ubiquitination, Myc-IRF3 was transfected into HEK293 cells together with Flag-TRIM26. Co-IP showed that the level of IRF3 ubiquitination was markedly increased in the presence of TRIM26 expression plasmid and MG-132 (Fig. 3C). Notably, two TRIM26 mutants in the RING-finger domain (C16A and C16/36A) lost the ability to promote IRF3 polyubiquitination (Fig. 3C), indicating TRIM26 promotes IRF3 ubiquitination though the RING-finger domain. Consistent with the inability of C16A to promote IRF3 ubiquitination, TRIF- and TBK1-induced IFN-β promoter activation was not affected by TRIM26 C16A (S2A Fig.). Furthermore, TRIM26 C16A did not facilitate VSV replication compared to WT TRIM26 (S2B Fig.). *In vitro* binding and ubiquitination assays demonstrated that TRIM26 could directly interact with IRF3 and promote IRF3 ubiquitination. Importantly, TRIM26-mediated IRF3 ubiquitination was dependent on the RING-finger domain because IRF3 ubiquitination induced by TRIM26 C16A was greatly attenuated compared to that induced by WT TRIM26 (Fig. 3D). To further confirm TRIM26-induced IRF3 ubiquitination, primary peritoneal macrophages were transfected with TRIM26 siRNA, and IRF3 ubiquitination was measured after SeV infection and LPS stimulation. SeV infection and LPS stimulation induced IRF3 ubiqutination (Fig. 3E). However, knockdown TRIM26 expression with siRNA substantially attenuated SeV- and LPS-induced IRF3 ubiquitination (Fig. 3E).

To investigate the form of polyubiquitin chains linked to IRF3, WT HA-Ubiquitin and its mutants K48 and K63, which has only one lysine residue in ubiquitin at position 48 and 63, respectively, were used. TRIM26-induced IRF3 ubiquitination could be easily detected in WT HA-Ub and K48 transfected cells (Fig. 3F). While, there is much less IRF3 ubiquitination in K63 transfected cells (Fig. 3F), suggesting that TRIM26 mainly conjugates K48-linked polyubiquitin chains to IRF3. K48-linked ubiquitination often leads to the degradation of target proteins by the 26S proteasome. Consistent with these observations, TRIM26-induced degradation of IRF3 was reversed by proteasome inhibitor MG-132, but not by lysosome inhibitor Chloroquine (Fig. 3G, lane 5 vs. lane 3 and 6). Importantly, TRIM26 C16A could not promote the degradation of IRF3 compared to WT TRIM26 (Fig. 3G, lane 4 vs. lane 3).

Previous studies have demonstrated that K70 and K87 are the two main ubiquitination sites in IRF3 [28]. To investigate whether TRIM26 promotes IRF3 ubiquitination through K70 and K87, IRF3 mutant K70/87A was transfected into HEK293 cells together with Flag-TRIM26. Compared to WT IRF3, TRIM26-induced IRF3 ubiquitination was decreased in IRF3 mutant K70/87A (Fig. 3H). Notably, TRIM26 did not induce the degradation of IRF3 mutant K70/87A compared to WT IRF3 (Fig. 3H, input, lane 3 vs. lane 4). All together, these data demonstrated that TRIM26 interacts with and promotes K48-linked polyubiqutination of IRF3 at K70/87, leading to IRF3 proteasomal degradation.

### Virus infection induces TRIM26 expression and nuclear translocation

**Fig 4:**
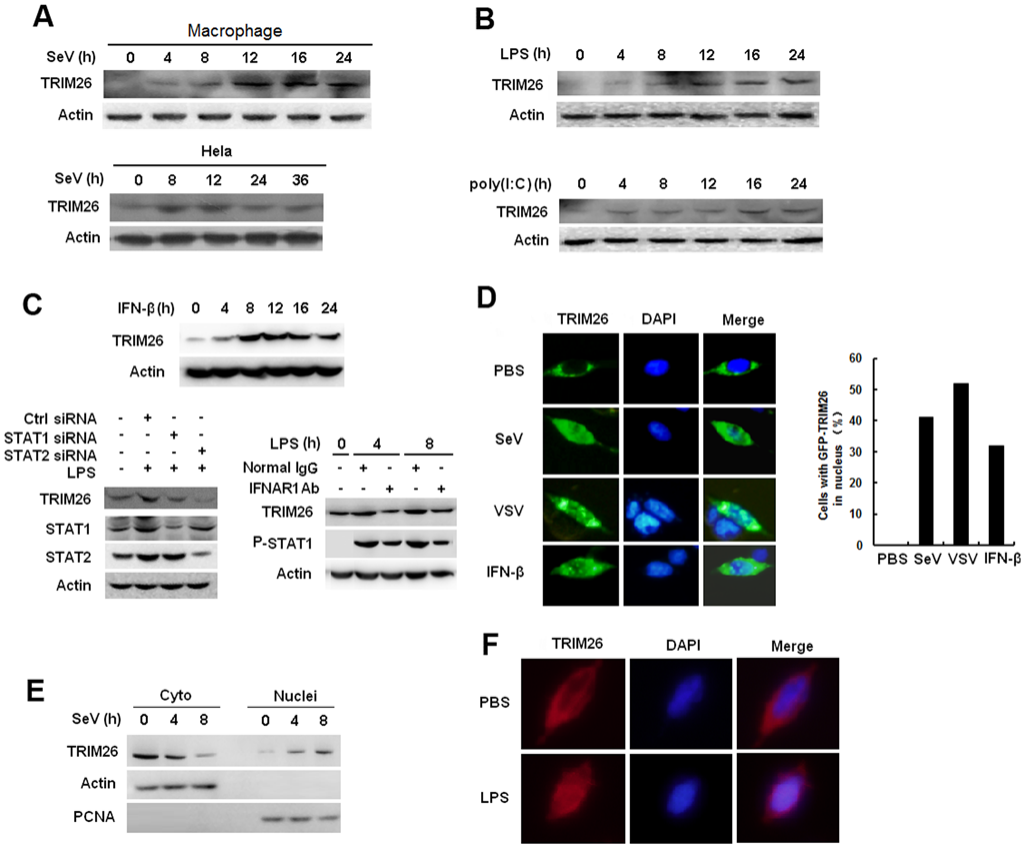
Virus infection induces TRIM26 expression and nuclear translocation. (A) Western blot analysis of TRIM26 protein expression in mouse primary peritoneal macrophages and Hela cells infected with SeV for indicated times. (B) Western blot analysis of TRIM26 protein expression in mouse primary peritoneal macrophages stimulated with LPS or poly(I:C) for indicated times. (C) Western blot analysis of TRIM26 protein expression in mouse peritoneal macrophages treated with mIFN-β for indicated times (left). Western blot analysis of TRIM26 protein expression in mouse peritoneal macrophages transfected with STAT1 siRNA, STAT2 siRNA and control siRNA (right, upper panel), or pretreated with IFNR1 antibody and control antibody for 2 h (right, lower panel), then simulated with LPS for indicated times. (D) Fluorescent images of HEK293 cells transfected with GFP-TRIM26 and then stimulated with SeV (MOI 1), VSV (MOI 0.1) or IFN-β for 8 h. Nuclei were detected with DAPI (blue). (E) Western blot analysis of TRIM26 protein expression in nuclear and cytoplasmic fractions from Hela cells infected with SeV for 4 and 8 h. (F) Immunofluorescent images of mouse peritoneal macrophages stimulated with 100 ng/ml LPS for 10 h. Endogenous TRIM26 was analyzed by immunostaining with anti-TRIM26 antibody (red). Similar results were obtained from three independent experiments.

Western blot analysis showed that TRIM26 protein is strongly expressed in various organs including lung, thymus, liver, spleen, small intestine and brain, but not in heart and kidney (S3A Fig.). Sendai virus (SeV) infection, which activates the RLR signaling, increased TRIM26 protein expression in primary peritoneal macrophages and Hela cells (Fig. 4A). TRIM26 expression was also induced in primary peritoneal macrophages upon LPS and poly(I:C) stimulation, which activate TLR4 and TLR3, respectively (Fig. 4B). TRIM26 mRNA expression was also induced by LPS and poly(I:C) stimulation and SeV infection in macrophages (S3B Fig.). Scanning of the mice TRIM26 promoter sequence identified a putative ISRE sequence (GATTTCACTTTCC, -162 bp to-150 bp), indicating TRIM26 expression may rely on the transcription factors STAT1 and STAT2 downstream of IFN-β signaling. Indeed, IFN-β stimulation increased TRIM26 mRNA and protein expression (Fig. 4C, upper panel and S3B Fig.). Blockage of IFN-β signaling by antibody against IFN-β receptor 1 (IFNR1) also attenuated LPS-induced TRIM26 expression (Fig. 4C, lower panel, right). Transfection of STAT1 or STAT2 specific siRNA substantially attenuated LPS-induced TRIM26 expression (Fig. 4C, lower panel, left). To investigate the cellular localization of TRIM26, GFP-TRIM26 was constructed and transfected into HEK293 cells. TRIM26 showed diffused expression in the cytoplasm without stimulation. While, a considerable proportion of TRIM26 moved into nucleus after infection with SeV and VSV (Fig. 4D). Biochemical assays with cytoplasmic and nuclear proteins also confirmed the translocation of TRIM26 into nucleus after SeV infection in Hela cells (Fig. 1E). This translocation may be mediated by IFN-β because IFN-β alone could induce TRIM26 nuclear translocation in HEK293 cells (Fig. 4D). Similarly, LPS induced TRIM26 translocation into nucleus in HEK293 cells stably expressing TLR4 (S3C FigC). Endogenous TRIM26 was also translocated into nucleus upon LPS stimulation in peritoneal primary macrophages (Fig. 4F). All together, these data suggested that TRIM26 expression is induced upon viral infection, which also induces the nuclear translocation of TRIM26.

### TRIM26 promotes IRF3 degradation in nucleus

**Fig 5:**
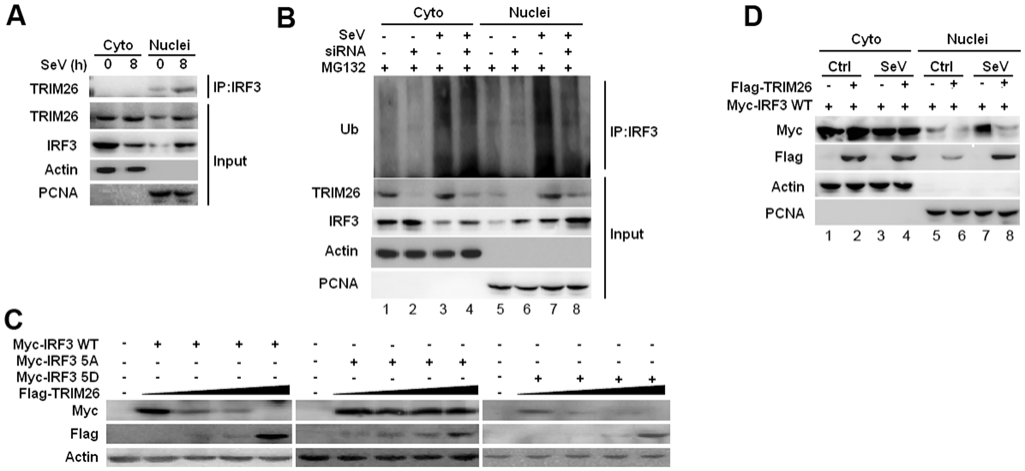
TRIM26 promotes IRF3 degradation in nucleus. (A) Nuclear and cytoplasmic fractions from RAW264.7 macrophages infected with SeV for 8 h or left uninfected were subjected to immunoprecipitation with anti-IRF3 antibody followed by western blot analysis with anti-TRIM26 antibody. (B) Nuclear and cytoplasmic fractions from RAW264.7 macrophages transfected with control siRNA (Ctrl) or TRIM26 siRNA (siRNA) and infected with SeV were subjected to immunoprecipitation with anti-IRF3 antibody followed by western blot analysis with anti-Ub antibody. (C) Western blot analysis of IRF3 expression in HEK293 cells transfected with expression plasmids for Myc-IRF3 WT, 5A or 5D and increased amount of Flag-TRIM26. (D) Western blot analysis of IRF3 expression in the cytoplasmic and nuclear fractions prepared from HEK293 cells, which were transfected with Myc-IRF3 WT and Flag-TRIM26 or control vector followed with SeV infection. Similar results were obtained from three independent experiments.

IRF3 normally shuttles between nucleus and cytoplasm, but is present dominantly in the cytoplasm prior infection. To investigate the location of TRIM26-mediated IRF3 ubiquitination and degradation, cytoplasmic and nuclear fractions were prepared from RAW264.7 macrophages after SeV infection. Co-IP showed that endogenous IRF3 interacted with TRIM26 in the nucleus, but not in the cytoplasm (Fig. 5A). SeV infection mainly promoted IRF3 ubiquitination in the nucleus in RAW264.7 cells and HEK293 cells (S4A and S4B Figs.). TRIM26 siRNA knockdown attenuated IRF3 ubiquitination in the nucleus (lane 8 vs. lane 7), while the level of IRF3 ubiquitination in the cytoplasm kept constant (Fig. 5B, lane 4 vs. lane 3), indicating that TRIM26 mainly interacts with and promotes IRF3 ubiquitination in the nucleus.

To investigate whether TRIM26 targets active IRF3 in nucleus, IRF3 WT and mutants 5D and 5A were transfected into HEK293 cells together with Flag-TRIM26. TRIM26 was found to degrade IRF3 WT and 5D in a dose dependent manner (Fig. 5C). In contrast, IRF3 5A could not be degraded by TRIM26 in the same settings (Fig. 5C). Notably, IRF3 5A was not degraded by TRIM26 even under the condition of infection with SeV and VSV (S4C Fig.). To investigate where IRF3 was degraded by TRIM26 in nucleus, cytoplasmic and nuclear fractions were prepared from IRF3 and TRIM26 or control vector transfected cells after infection with SeV or left uninfected. A considerable proportion of IRF3 was translocated into nucleus upon IRF3 overexpression without SeV infection (Fig. 5D, lane 5). SeV infection induced further nuclear translocation of IRF3 (Fig. 5D, lane 7). Overexpression of TRIM26 promoted the degradation of nuclear IRF3 (Fig. 5D, lane 6 and 8), but not the cytoplasmic IRF3 (Fig. 5D, lane 2 and 4). Similar to WT IRF3, a considerable proportion of 5D was translocated into nucleus upon overexpression (S4D Fig.). However, a very small proportion of IRF3 5A was translocated into nucleus compared to WT IRF3 and 5D (S4D Fig.). Overexpression of TRIM26 also promoted the degradation of IRF3 5D in nucleus (S4D Fig, lane 12), but not in cytoplasm (lane 10). IRF3 5A was not degraded in both cytoplasm and nucleus (S4D Fig.). These data suggested that TRIM26 mainly promotes the degradation of active form IRF3 in nucleus.

### Nuclear translocation promotes TRIM26-mediated IRF3 degradation

**Fig 6:**
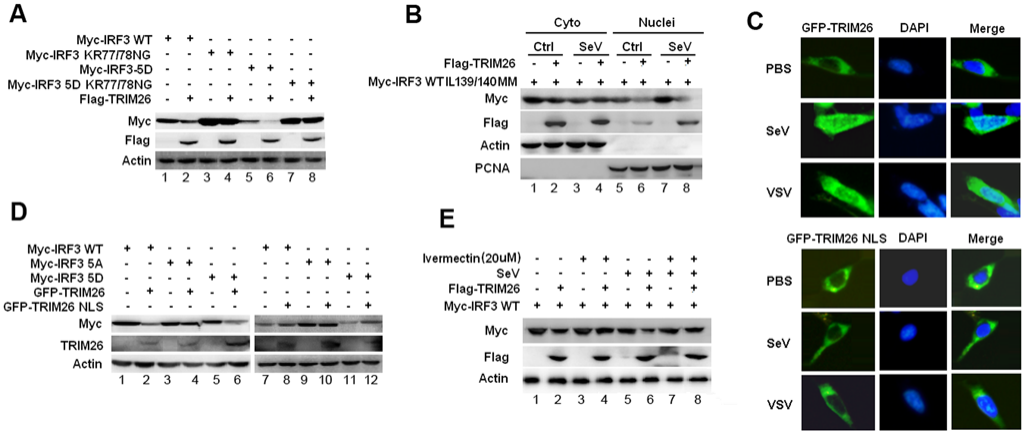
Nuclear translocation promotes TRIM26-mediated IRF3 degradation. (A) Western blot analysis of IRF3 expression in HEK293 cells transfected with expression plasmids for Myc-IRF3 WT, KR77/78NG, 5D or 5D KR77/78NG and Flag-TRIM26. (B) Western blot analysis of IRF3 IL139/140MM expression in the cytoplasmic and nuclear fractions prepared from HEK293 cells, which were transfected with Myc-IRF3 IL139/140MM and Flag-TRIM26 or control vector followed with SeV infection. (C) Fluorescent images of HEK293 cells transfected with GFP-TRIM26 or GFP-TRIM26 NLS plasmid and infected with SeV and VSV. Nuclei were detected with DAPI (blue). (D) Western blot analysis of IRF3 expression in HEK293 cells transfected with expression plasmids for Myc-IRF3 WT, 5A or 5D and GFP-TRIM26 or GFP-TRIM26 NLS. (E) Western blot analysis of IRF3 expression in HEK293 cells transfected with Myc-IRF3 and Flag-TRIM26 pretreated with 20μM Ivermectin for 2 h. Similar results were obtained from three independent experiments.

The localization of IRF3 is mediated by both the nuclear localization signal (NLS) and nuclear exporter signal (NES) [29]. IRF3 mutant KR77/78NG in the NLS lost the ability to translocate into nucleus after viral infection [29]. To investigate TRIM26-mediated IRF3 degradation is really in nucleus, IRF3 WT and IRF3 KR77/78NG were transfected into HEK293 cells together with TRIM26 expression plasmid. Compared to WT IRF3, the protein level of IRF3 KR77/78NG was greatly increased (Fig. 6A, lane 3 vs. lane 1). Importantly, KR77/78NG could not be degraded by TRIM26 (Fig. 6A, lane 4 vs. lane 3). TRIM26 also could not degrade KR77/78NG mutant even in the active form IRF3 5D (Fig. 6A, lane 8 vs. lane 7), and the protein level of IRF3 5D-KR77/78NG was greatly increased compared to that of IRF3 5D (Fig. 6A, lane 7 vs. 5). Consistent with the inability to degrade IRF3 KR77/78NG, Flag-TRIM26 was not interacted with IRF3 KR77/78NG, and 5D KR77/78NG (S5A Fig.). IRF3 mutant ΔDBD with the deletion of the DNA-binding domain (aa 1–115), which covers the NLS, could not be degraded by TRIM26 (S5B Fig.).

Deletion of the NES resulted in the nuclear accumulation of IRF3. To further confirm IRF3 was degraded in the nucleus, IRF3 NES mutant IL139/140MM was used. A large amount of IRF3 IL139/140MM was accumulated in the nucleus especially after SeV infection (Fig. 6B, lane 5 and 7). Western blot analysis of cytoplasmic and nuclear fractions showed that IRF3 IL139/140MM was efficiently degraded by TRIM26 in nucleus (Fig. 6B, lane 6 and 8), but not in the cytoplasm (Fig. 6B, lane 2 and 4). Furthermore, TRIM26 was found to degrade IRF3 5D-IL139/140MM, but not the 5A-IL139/140MM (S5C Fig.). Consistently, TRIM26 was found to interact with IRF3 WT IL139/140MM and 5D-IL139/140MM, but not with 5A-IL139/140MM (S5A Fig.). These data demonstrated that IRF3 nuclear translocation is required for TRIM26-induced degradation.

TRIM26 was translocated into nucleus after viral infection or TLR stimulation (Fig. 1). A recent study also demonstrated that TRIM26 was recruited to histone modifier Jmjd3 to mediate PHF20 ubiquitination and degradation [30]. These data indicate a functional NLS may be present in TRIM26 to facilitate its nuclear translocation. Indeed, PSORT program predicated a putative NLS rkkfwvgkpiarvvkkk between aa 265 and aa 281 in TRIM26. To investigate whether this NLS is responsible for TRIM26 nuclear translocation, the conserved amino acids (RKK and KKK) were mutated to arginine to give rise TRIM26 NLS mutant TRIM26-M-NLS. Fluorescent microscopy showed that TRIM26-M-NLS lost the ability to translocate into the nucleus after SeV and VSV infection (Fig. 6C). Similar to Flag-TRIM26, GFP-TRIM26 overexpression efficiently promoted the degradation of WT IRF3 and 5D (Fig. 6C, lane 1 and 6), but, not of 5A (Fig. 6D, lane 4). Whereas, GFP-TRIM26-M-NLS lost the ability to degrade both WT IRF3 and 5D (Fig. 6D, lane 8 and 12), indicating TRIM26 nuclear localization is required for IRF3 degradation.

To directly confirm nuclear translocation of IRF3 and TRIM26 is required for IRF3 degradation, nuclear import inhibitor Ivermectin was used. Ivermectin treatment prevented the nuclear translocation of IRF3 after SeV infection (S5D Fig.). While, IRF3 phosphorylation was not impaired by Ivermectin treatment (S5E Fig.). Notably, inhibition of IRF3 nuclear translocation by Ivermectin abolished TRIM26-induced IRF3 degradation in SeV-infected cells and uninfected cells (Fig. 6D, lane 4 Vs. lane 2 and lane 8 Vs. lane 5). Taken together, these results suggested that TRIM26 mediates ubiquitination and degradation of active IRF3 in the nucleus.

### Impaired IFN-β signaling and antiviral responses in TRIM26 transgenic mice

To investigate the physiological function of TRIM26, TRIM26-transgenic mice (TRIM26-Tg mice) was established. The transgenic mice were identified by PCR assays of genomic DNA from tails of transgenic mice (S6A Fig.). TRIM26-Tg mice are viable, normal in size and without gross physiological or behavioral abnormalities (data now shown). The expression of TRIM26 mRNA and protein in the thymus from the TRIM26-Tg mice was higher than that of the WT littermate (S6B Fig.). Similarly, the expression of TRIM26 mRNA and protein in peritoneal macrophages from TRIM26-Tg mice was higher than that from WT mice before and after SeV infection (S6C Fig.). Consistent with the data of TRIM26-mediated IRF3 degradation, SeV-induced IRF3 phsophorylation was reduced in macrophages from TRIM26-Tg mice, compared to that from WT mice (S6D Fig.). Notably, the level of total IRF3 was also decreased in macrophages from TRIM26-Tg mice. Consistent with function of TRIM26 to mediate IRF3 ubiquitination, more IRF3 ubiquitination was detected in macrophages from TRIM26-Tg mice after SeV infection compared to that from WT mice (S6E Fig.).

**Fig 7:**
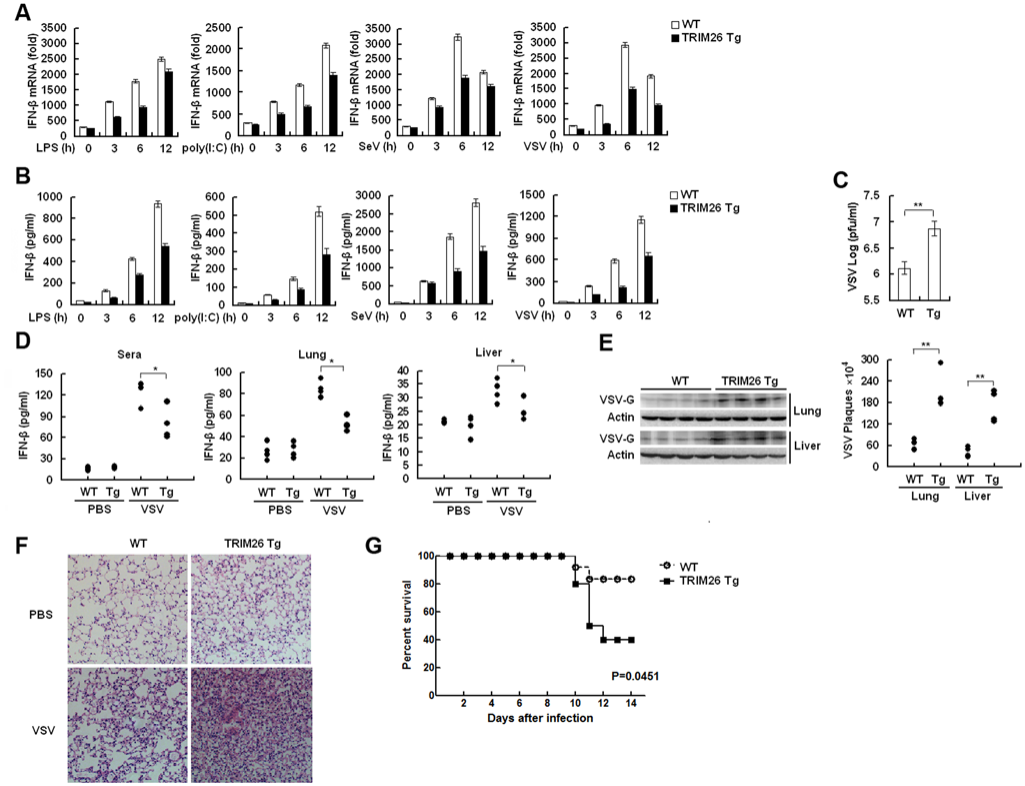
Impaired IFN-β signaling and antiviral responses in TRIM26 transgenic mice. (A–B) Expression of IFN-β mRNA and secretion of IFN-β protein measured by QRT-PCR and ELISA, respectively, in peritoneal macrophages from TRIM26-Tg and WT mice stimulated with LPS and poly(I:C) or infected with SeV and VSV for indicated times. (C) Peritoneal macrophages from TRIM26-Tg and WT mice were infected with VSV (MOI, 0.1) for 12 h. Supernatants were analyzed for VSV titers with standard plaque assays. (D) ELISA analyses of IFN-β production in sera, lung or liver from TRIM26-Tg and WT mice intravenously infected with VSV for 72 h. (n = 4 mice per group). (E) TRIM26-Tg and WT mice were infected with VSV as in D. VSV titers in lung and liver were determined by standard plaque assays (right). Expression of VSV G protein was analyzed by western blot with VSV-G antibody (left). (F) Hematoxylin and eosin staining of lung tissue sections from TRIM26-Tg and WT mice infected with VSV as in e. (G) Survival of TRIM26-Tg and WT mice (n = 10) infected with VSV (4×10^7^ pfu/mouse). Data are representative of three independent experiments (mean ± S.D. of triplicates in A-D).

Primary peritoneal macrophages from TRIM26-Tg and WT mice were prepared and stimulated with LPS and poly(I:C) or infected with SeV and VSV, the expression of IFN-β mRNA and secretion of IFN-β protein was measured by quantitative RT-PCR and ELISA, respectively. After stimulation with LPS and poly(I:C) or infection with SeV and VSV, macrophages from TRIM26-Tg mice showed less IFN-β expression and secretion, compared to the macrophages from WT mice (Fig. 7A and B). VSV replication in macrophages from TRIM26-Tg mice was greatly increased compared to that from WT mice (Fig. 7C). To test the importance of TRIM26 *in vivo*, TRIM26-Tg mice and control WT littermates were infected with VSV, and the antiviral immune responses were examined. The amount of IFN-β protein induced by VSV infection was much less in sera, lung and liver of TRIM26-Tg mice than that of VSV-infected WT mice (Fig. 7D). In accordance with reduced IFN-β production, VSV replication in the lungs and livers of TRIM26-Tg mice was higher than WT controls (Fig. 7E). HE staining showed that severe infiltration of immune cells and injury were observed in the lungs of TRIM26-Tg mice, compared to that of WT mice after virus infection (Fig. 7F). Moreover, TRIM26-Tg mice were more susceptible to VSV infection than WT mice (Fig. 7G). These data suggested that TRIM26 is an important negative regulator of IFN-β production and antiviral immune responses, therefore TRIM26-transgenic mice have impaired antiviral response.

## Discussion

Several studies have demonstrated that phosphorylated IRF3 underwent ubiquitination and proteasomal degradation after infection with Sendai virus [23], [25]. However, the identity of the E3 ubiquitin ligase that is responsible for the ubiquitination of nuclear IRF3 is not defined. Here we have provided evidence to show that TRIM26 is an E3 ubiquitin ligase to promote the ubiquitination and degradation of nuclear IRF3. 1) TRIM26 interacted with IRF3 in the nucleus after TLR4 activation and SeV infection in macrophages. Furthermore, TRIM26 was found to interact with WT IRF3 and constitutive phosphorylation active mutant 5D, but not with phosphorylation deficient mutant 5A in co-transfection assays. 2) TRIM26 knockdown by siRNA mainly attenuated SeV-induced IRF3 ubiquitination in nucleus, but, has little effect for the cytoplasmic IRF3 ubiquitination. 3) TRIM26 was found to degrade WT IRF3 and 5D, but not 5A. Further analysis of cytoplasmic and nuclear fractions demonstrated that WT IRF3 and IRF3 5D were degraded by TRIM26 in nucleus, not in the cytoplasm. 4) IRF3 mutants KR77/78NG and 5D-KR77/78NG with mutation in the NLS could not interact with and be degraded by TRIM26. 5) TRIM26 mutant in the NLS, which did not move into nucleus, lost the ability to degrade IRF3. 6) Chemical inhibitor to inhibit IRF3 and TRIM26 nuclear translocation abolished TRIM26-induced IRF3 degradation. All together, these data indicated that active IRF3 was ubiquitinated and degraded by TRIM26 in nucleus.

Normally, IRF3 shuttles between nucleus and cytoplasm and a small portion of IRF3 translocates into nucleus [29]. This small portion of IRF3 may be targeted by TRIM26 to prevent the IFN-β production in normal conditions. This may explain why WT IRF3 was degraded without virus infection and the level of total IRF3 was decreased in TRIM26-Tg mice. In supporting this claim, we found the expression level of IRF3 mutant KR77/78NG in the NLS was much higher than the WT IRF3 in the presence of TRIM26. IRF3 WT and IRF3 5A have the same constitutive shuttling ability between cytoplasm and nucleus, while TRIM26 only promoted WT IRF3 degradation, but not 5A. Clearly, besides the constitutive shuttling between cytoplasm and nucleus, other factors also contribute to TRIM26-mediated IRF3 degradation. We found a considerable proportion of WT IRF3 was translocated into nucleus upon overexpression, which was similar to IRF3 5D. While this was not the case for IRF3 5A, a very small portion of IRF3 5A was transfected into nucleus. The different ability of nuclear translocation between IRF3 WT and 5A may be caused by IRF3 phosphorylation because phosphorylation is the prerequisite for IRF3 nuclear translocation. In fact, overexpression of IRF3 WT could induce IFN-β production and activation of IFN-β promoter in our system, indicating partial of WT IRF3 was phosphorylated and translocated into nucleus upon overexpression. Thus, IRF3 WT was targeted by TRIM26 for degradation because of its ability to translocate into nucleus, while 5A could not be targeted by TRIM26 because of the inability to move into nucleus due to the deficiency of phosphorylation. All together, our data indicated IRF3 phosphorylation and nuclear translocation are required for TRIM26-mediated degradation.

Several E3 ligases have been demonstrated to promote IRF3 ubiquitination and degradation. For example, RAUL, a HECT domain-containing E3 ligase, has been shown to promote ubiquitination and proteasomal degradation of IRF3 and IRF7 [31]. Although RAUL could degrade IRF3 and IRF7 in both nucleus and cytoplasm, RAUL promotes the degradation of the phosphorylation deficient mutant of IRF3 and IRF7, indicating RAUL may mainly promote the degradation of un-active form IRF3 and IRF7. E3 ubiquitin ligase RBCK1 has also shown to promote the degradation and ubiquitination of IRF3 [32]. Interestingly, RBCK1 specifically destabilizes IRF3, but not IRF7, which is different from TRIM26. We found IRF7 was degraded by TRIM26 overexpression (S7A Fig.). IRF9 was also degraded by TRIM26 (S7B Fig.). The E3 ligase Ro52 also known as TRIM21, has reported to target IRF3 for polyubiquitination and proteasomal-mediated degradation [33]. Although TRIM21 interacted with IRF3 after poly(I:C) stimulation in HEK293 cells, where IRF3 was ubiquitinated and whether the active form of IRF3 was targeted are not investigated in this report. We found TRIM21 promoted the degradation of IRF3 WT, 5A and 5D (S8A Fig.). Cullin 1-based ubiquitin ligase has been reported to promote the ubiquitination and degradation of phosphorylated IRF3 [34]. However, siRNA knockdown of Cullin 1 expression did not affect TRIM26-mediated IRF3 degradation (S8B Fig.). These data suggested that there is no competition between Cullin 1 and TRIM26. Therefore, TRIM26 is an essential E3 ubiquitin ligase specifically targeting active IRF3 for degradation in nucleus.

There are more than 70 members in the TRIM protein family. Accumulating results have confirmed that TRIM proteins play an essential role in the regulation of innate immune response by regulating the signal transduction mediated by PRRs [35]-[38]. Using TRIM26 transgenic mice, we demonstrated that overexpression of TRIM26 greatly attenuated IFN-β production in primary peritoneal macrophages after virus infection and TLR activation. Expression of IFN-β also greatly decreased in TRIM26 transgenic mice after virus infection *in vivo*. As a consequence, TRIM26 transgenic mice showed an impaired ability to inhibit virus replication and were more susceptible to virus infection. These *in vitro* and *in vivo* data strongly suggested that TRIM26 is a negative regulator for IFN-β production and antiviral immune responses. However, whether TRIM26 has redundancy to regulate IFN-β production with other TRIM family members requires the construction of TRIM26 deficient mice.

Ubiquitination-mediated degradation of transcription factors is now recognized as an efficient way to regulate transcription and terminate cellular signaling [39], [40]. Here we reported that TRIM26 negatively regulates activation of IRF3 transcription factor downstream of various signaling pathway including TLR signaling, RLR signaling and DNA-mediated signaling. Thus, our results identified a novel pathway to limit virus-induced signaling, inflammation and tissue injury after infection. At the same time, we demonstrated that virus infection increased TRIM26 expression and nuclear translocation, which promoted the ubiquitination and degradation of nuclear IRF3 leading to decreased IFN-β production and antiviral responses. Therefore, virus may induce TRIM26 expression to modulate the IRF3 activation and IFN-β production to facilitate their evasion of the innate immune system.

In summary, we identified a novel function for TRIM26 to negatively regulate IFN-β production and antiviral responses by targets nuclear IRF3 for ubiquitination and degradation. Given the pathological role of IFN-β in SLE and other autoimmune diseases, TRIM26 may be used as a therapeutic target to limit IFN-β overproduction to prevent and cure these diseases. Moreover, TRIM26 may also be used as a target for drug design to prevent the viral invasion of the innate immune responses.

## Materials and Methods

### Mice, cells and reagents

C57BL/6J mice for preparation of peritoneal macrophages were obtained from Joint Ventures Sipper BK Experimental Animal (Shanghai, China). Mouse macrophage cell line RAW264.7, human HEK293 and Hela cells were obtained from American Type Culture Collection (Manassas, VA). HEK293/TLR4 and TLR3 cell lines were obtained from Invivogen (San Diego, CA, USA). Mouse primary peritoneal macrophages were prepared as described [41]. The cells were cultured at 37°C under 5% CO2 in DMEM supplemented with 10% FCS (Invitrogen-Gibco), 100 U/ml penicillin, and 100 μg/ml streptomycin. MG132, Chloroquine, JSH-23, and LPS (Escherichia coli, 055:B5) were purchased from Sigma (St. Louis, MO). poly(I:C), poly(dA:dT) and ISD were purchased from Invivogen (San Diego, CA, USA). LPS, poly(I:C), poly(dA:dT) and ISD were used at a final concentration of 100 ng/ml, 10 μg/ml, 2μg/ml and 10μg/ml, respectively. Ivermectin were purchased from Sigma and used at final concentration of 20μM. IFN-β was from PeproTech (Rocky Hill, New Jersey, USA) and used at a final concentration of 1000 U/ml. The antibodies specific for HA, Ub, TRIM26, STAT1, STAT2, β-actin, GAPDH and protein G agarose used for immunoprecipitation were from Santa Cruz Biotechnology (Santa Cruz, CA). Antibody against mice IFNR1 was purchased from Leinco Technologies (St. Louis, MO). The antibodies specific to Myc, IRF3, TBK1, phospho-IRF3 and PCNA were from Cell Signaling Technology (Beverly, MA). Antibody specific to Cullin 1 was from OriGene Technologies (Rockville, MD). The antibody for Flag and VSV G protein were from Sigma (St. Louis, MO). Their respective horseradish peroxidase-conjugated secondary antibodies were purchased from Santa Cruz Biotechnology (Santa Cruz, CA). Sendai virus was purchased from China Center for Type Culture Collection (Wuhan University, China). Vesicular stomatitis virus (VSV) was provided by Professor Hong Meng (Institute of Basic Medicine, Shandong Academy of Medical Sciences, China).

### Sequences, plasmid constructs and transfection

pCMV6-Flag-TRIM26 expression plasmid was purchased from OriGene (Rockville, MD). GFP-TRIM26 was generated by subcloning of TRIM26 coding sequence into pEGFP-N1 vector (CloneTech, CA). Expression plasmid for cGAS was constructed by subcloning of the coding sequence into corresponding vectors. Mutant plasmids for TRIM26 and IRF3 including TRIM26 C16A, C16/36A, GFP-TRIM26-NLS, IRF3 5D, 5A, KR77/78NG, IL139/140MM, ΔDBD and K70/87R were generated using the KOD-Plus-Mutagenesis kit (Toyobo, Osaka, Japan). All constructs were confirmed by DNA sequencing. HA-TRIM21 expression plasmid was provided by Dr. Chen Wang (Shanghai Institutes for Biological Sciences, Shanghai, China). Flag-IRF7 and IRF9 plasmids were provided by Dr. Alexander Espinosa (Department of Medicine, Weill Cornell Medical College). IFN-β reporter plasmid was provided by Prof. Xuetao Cao (Secondary Military Medical University, China) [42]. The ISRE reporter plasmid was provided by Prof. Hong-bing Shu (Wuhan University, China). Other plasmids used in this study were described previously [43]. For transient transfection of plasmids into RAW264.7 cells, jetPEI reagents were used (Polyplus-transfection). For transient transfection of plasmids into HEK293 cells, lipofectamine 2000 reagents were used (Invitrogen). For transient silencing, duplexes of small interfering RNA were transfected into cells with the Geneporter 2 Transfection Reagent (GTS, San Diego) according to the standard protocol. Target sequences for transient silencing were 5’-CCAAGGACUUCGCCAACAA-3’(siRNA 1) and 5’-GAAGUUCUGGAUUGGGAAA-3’ (siRNA 2) for TRIM26, ‘scrambled’ control sequences were 5’-UUCUCCGAACGUGUCACGU-3’. STAT1 siRNA and STAT2 siRNA were obtained from Santa Cruz Biotechnology (Santa Cruz, CA). Cullin 1 siRNA were from OriGene Technologies (Rockville, MD).

### ELISA

The concentrations of IFN-β in culture supernatants, sera, liver and lung were measured by ELISA Kits (R&D Systems, Minneapolis, MN).

### RNA quantitation, immunoprecipitation and western blot analysis

Total RNA was extracted with TRIzol reagent according to the manufacturer’s instructions (Invitrogen). Specific primers used for RT-PCR assays were 5’- ATTCTGAACCACTTGAACACCC-3’, 5’- ATTCCGCCACAATGTACTGC-3’ for mTRIM26, 5’-AGTTACACTGCCTTTGCC-3’, 5’-GTTGAGGACATCTCCCAC-3’ for mIFN-β, and 5’-CAACAAGTGTCTCCTCCAAAT-3’, 5’-TCTCCTCAGGGATGTCAAAG-3’ for hIFN-β. For immunoblot analysis, cells or tissues were lysed with M-PER Protein Extraction Reagent (Pierce, Rockford, IL) supplemented with a protease inhibitor ‘cocktail’. Nuclear proteins and cytoplasmic proteins were extracted by NE-PER Protein Extraction Reagent (Pierce) according to the manufacturer’s instructions. Protein concentrations in the extracts were measured with a bicinchoninic acid assay (Pierce, Rockford, IL) and were made equal with extraction reagent. For immunoprecipitation (IP), whole-cell extracts were collected 36 h after transfection and were lysed in IP buffer containing 1.0% (vol/vol) Nonidet P 40, 50 mM Tris-HCl, pH 7.4, 50 Mm EDTA, 150 mM NaCl, and a protease inhibitor ‘cocktail’ (Merck). After centrifugation for 10 min at 14,000g, supernatants were collected and incubated with protein G Plus-Agrose Immunoprecipitation reagent (Santa Cruz) together with 1 μg corresponding antibodies. After 6 h of incubation, beads were washed five times with IP buffer. Immunoprecipitates were eluted by boiling with 1% (wt/vol) SDS sample buffer. For western blot analysis, immunoprecipitates or whole-cell lysates were loaded and subjected to SDS-PAGE, transferred onto nitrocellulose membranes, and then blotted with specific antibodies. The levels of phosphorylated IRF3 were quantified by measuring the band densitometry, which are normalized to the band densitometry of Actin.

### Assay of luciferase activity

Luciferase activity was measured with the Dual-Luciferase Reporter Assay system according to the manufacturer’s instructions (Promega) as described (43). Data were normalized for transfection efficiency by division of firefly luciferase activity with that of renilla luciferase.

### Ubiquitination assays

For analysis of the ubiquitination of IRF3 in HEK293 cells, HEK293 cells were transfected with Myc-IRF3, HA-Ub (WT) or HA-Ub mutants and Flag-TRIM26 WT or mutants, and then whole-cell extracts were immunoprecipitated with anti-Myc and analyzed by immunoblot with anti-HA antibody. For analysis of the ubiquitination of IRF3 in macrophages, macrophages were infected with SeV, then whole-cell extracts or cytoplasmic and nuclear fractions were immunoprecipitated with anti-IRF3 and analyzed by immunoblot with anti-Ub antibody.

### *In vitro* binding and ubiquitination assay

IRF3, TRIM26 WT and C16/36A mutant proteins were expressed with a TNT Quick Coupled Transcription/Translation System (Promega) according to the instructions of the manufacturer. Binding assays were performed by mixing TRIM26 and IRF3 together, followed by IP with TRIM26 antibody and WB with IRF3 antibody. Ubiquitination was analyzed with an ubiquitination kit (Boston Biochem) following protocols recommended by the manufacturer.

### VSV plaque assay and detection of virus replication

VSV plaque assay was performed as described [43]. Hela cells or macrophages (2×10^5^) were transfected with the indicated plasmids or TRIM26 siRNA for 36 h prior to VSV infection (MOI of 0.1). At 1 h after infection, cells were washed with PBS for three times and then medium was added. The supernatants were harvested at 24 h after washing. The supernatants were diluted and then used to infect confluent HEK293 cells cultured on 24-well plates. At 1 h postinfection, the supernatant was removed, and 3% methylcellulose was overlayed. At 3-days postinfection, overlay was removed; cells were fixed with 4% formaldehyde for 20 min, and stained with 0.2% crystal violetin. Plaques were counted, averaged, and multiplied by the dilution factor to determine viral titer as Pfu/ml. Total cellular RNA was extracted and VSV RNA replicates were examined by Quantitative RT-PCR as described (43). Primers for VSV were as follows: 5’-ACGGCGTACTTCCAGATGG-3’ (sense) and 5’-CTCGGTTCAAGATCCAGGT-3’ (antisense).

### Immunofluorescence staining and microscopy

HEK293 cells transiently transfected with plasmids encoding GFP-TRIM26 or Flag-TRIM26 were cultured on coverslips for 48 hours. Then the cells were infected with SeV or VSV or stimulated with IFN-β or LPS. For the cells transfected with GFP-TRIM26, cells were examined directly with an Olympus IX71 fluorescence microscope (Olympus Co., Tokyo, Japan). For HEK293/TLR4 cells transfected with Flag-TRIM26 or macrophages, cells were sequentially immunostained first with antibody against Flag or TRIM26 antibody, and then with proper Alexa Fluor 568-conjugated secondary Antibody (Molecular Probes, Invitrogen). DAPI (4’, 6’-diamidino-2-phenylindole hydrochloride; Molecular Probes, Invitrogen) was used to stain nuclei.

### Transgenic mice

Transgenic founder mice expressing TRIM26 gene (TRIM26-Tg mice) were generated by Cyagen Biosciences Inc. (Guangzhou, Guangdong, China) in the FVB background and mated with WT FVB mice to produce mice used in all the experiments. Expression of TRIM26 was under the control of EF1a promoter (elongation factor 1a promoter). TRIM26-Tg mice produced viable offspring. The transgenic mice were identified by PCR of genomic DNA from tails with the following primers: 5’-ACGTAAACGGCCACAAGTTC-3’ (sense), 5’-GATCTTGAAGTTCACCTTGATGC-3’ (antisense). All mice used are 6 to 8 months of age.

### VSV infection of mice

TRIM26-Tg or WT mice (female, 6–8 weeks old) were intravenously infected with VSV (5×10^7^ pfu per mouse) as described [44]. The virus titres in lung and liver were determined by standard plaque assays and by measurement of VSV V protein with VSV-G antibody. For the survival experiments, mice were monitored for survival after VSV infection. Lungs from control or virus-infected mice were dissected, fixed in 10% phosphate-buffered formalin, embedded into paraffin, sectioned, stained with hematoxylin-eosin solution and examined by light microscopy for histologic changes.

### Statistical analysis

All data are presented as mean ± S.D. of three or more experiments. Statistical significance was determined with the two-tailed Student’s t-test, with a P value of less than 0.05 considered statistically significant.

### Ethics statement

All animal experiments were undertaken in accordance with the National Institute of Health Guide for the Care and Use of Laboratory Animals, with the approval of the Scientific Investigation Board of Medical School of Shandong University, Jinan, Shandong Province, China (Permit number: 201401039).

### Proteins accession numbers

The accession numbers in the UniProtKB/SwissProt database for the proteins in the manuscript are followed: TRIM26, Q12899; IRF3, Q14653; cGAS, Q8N884; IFN-β, P01574; RIG-I, O95786; MAVS, Q7Z434; TRIF, Q8IUC6; TBK1, Q9UHD2; STING, Q86WV6; STAT1, P42225; STAT2, Q6P1X8; VSV-G, P04882.

## Supporting Information

S1 Fig TRIM26 attenuates VSV-induced IFN-β production. (A) Expression of IFN-β mRNA in Hela cells transfected with Flag-TRIM26 expression plasmid or control vector (Ctrl) followed with infection with VSV for indicated times. (B) Expression of IFN-β mRNA in mice peritoneal macrophages transfected with TRIM26 siRNA or control siRNA (Ctrl) followed with infection with VSV for indicated times. Data are representative of three independent experiments (mean ± S.D. of duplicates in A and B).
(TIF)

S2 Fig TRIM26 C16A mutant has impaired function. (A) HEK293 cells were transfected with expression plasmids for TRIF or TBK1, along with IFN-β reporter plasmid and TRIM26 WT or C16A plasmid, and analyzed luciferase activity. (B) HEK293 cells were transfected with expression plasmids for TRIM26 WT, C16A or control vector (Ctrl). 24 h later, cells were further transfected with poly(I:C) or left untreated. 18 h after poly(I:C) transfection, cells were infected with VSV (MOI, 0.1), and the supernatants were harvested at 12 h post-infection. Supernatants were analyzed for VSV titers with standard plaque assays. Data are representative of three independent experiments (mean ± S.D. of quadruplicates in A and triplicates in B).
(TIF)

S3 Fig Characterization of TRIM26 expression. (A) Western blot analysis of TRIM26 protein expression in different mouse tissues. (B) RT-PCR analysis of TRIM26 mRNA expression in peritoneal macrophages stimulated with LPS, poly(I:C), IFN-β or infected with SeV for indicated times. (C) Immunofluorescent images of HEK293/TLR4 cells transfected with Flag-TRIM26 plasmid and then stimulated with LPS for 1 h. Flag-tagged TRIM26 was analyzed by immunostaining with anti-Flag antibody (red). Data are representative of three independent experiments.
(TIF)

S4 Fig TRIM26 promotes IRF3 ubiquitination and degradation in nucleus. (A) Nuclear and cytoplasmic fractions prepared from RAW264.7 cells after infection with SeV were subjected to immunoprecipitation with anti-IRF3 antibody followed by western blot analysis with anti-Ub antibody. (B) Nuclear and cytoplasmic fractions from HEK293 cells transfected with Myc-IRF3 plasmid followed by SeV infection were subjected to immunoprecipitation with anti-Myc antibody followed by western blot analysis with anti-Ub antibody. (C) Western blot analysis of Myc-IRF3 5A expression in HEK293 cells transfected with expression plasmid for Myc-IRF3 5A and Flag-TRIM26 followed with VSV or SeV infection. (D) Western blot analysis of IRF3 expression in cytoplasmic and nuclear fractions prepared from HEK293 cells transfected with Myc-IRF3 WT, 5A, 5D together with Flag-TRIM26. Similar results were obtained from three independent experiments.
(TIF)

S5 Fig Nuclear translocation promotes TRIM26-mediated IRF3 degradation. (A) Lysates from HEK293 cells transfected with Flag-TRIM26, IRF3 NLS mutants and NES mutants or control vector and followed with treatment with MG-132, were subjected to immunoprecipitation with anti-Myc antibody followed by western blot analysis with anti-Flag antibody. (B) Western blot analysis of the expression IRF3 mutant ΔDBD in HEK293 cells transfected with Myc-IRF3 ΔDBD and Flag-TRIM26 or control vector. (C) Western blot analysis of IRF3 expression in HEK293 cells transfected with Myc-IRF3 WT and various mutants in the NES. (D) Western blot analysis of IRF3 protein in cytoplasmic and nuclear fractions prepared from HEK293 cells pretreated with different concentrations of Ivermectin for 2 h, followed by SeV infection for 4 h. (E) Western blot analysis of phosphorylated IRF3 and total IRF3 protein in HEK293 cells pretreated with different concentrations of Ivermectin for 2 h, followed by SeV infection for 4 h. Similar results were obtained from three independent experiments.
(TIF)

S6 Fig Identification of TRIM26 transgenic mice. (A) TRIM26 transgenic mice were identified by PCR assays of genomic DNA from tails of mice. (B) RT-PCR and western blot analysis of the expression of TRIM26 mRNA and protein in the thymus from the TRIM26-Tg mice and WT mice identified in (A). (C) RT-PCR and western blot analysis of the expression of TRIM26 mRNA and protein in peritoneal macrophages from TRIM26-Tg mice and WT mice before and after SeV infection. (D) Western blot analysis of phosphorylated-IRF3, total IRF3 and TRIM26 in peritoneal macrophages from TRIM26-Tg and WT mice infected with SeV for indicated times. (E) Lysates prepared from peritoneal macrophages from TRIM26-Tg and WT mice pretreated with MG-132 for 2 h and then infected with SeV for indicated times, were subject to IP with IRF3 antibody followed by IB with Ubiquitin antibody. Data are representative of five (A) and three (B-E) independent experiments.
(TIF)

S7 Fig TRIM26 promotes the degradation of IRF7 and IRF9. Western blot analysis of IRF7 and IRF9 expression in HEK293 cells transfected with Flag-IRF7 (A) and Flag-IRF9 (B) together with Myc-TRIM26 or control vector. Similar results were obtained from three independent experiments.
(TIF)

S8 Fig TRIM21- and Cullin 1-mediate IRF3 degradation. (A) Western blot analysis of Myc-IRF3 expression in HEK293 cells transfected with expression plasmid for Myc-IRF3 WT, 5D and 5A together with HA-TRIM21 or control vector. (B) Cullin 1 specific siRNA or control siRNA were transfected into HEK293 cells, 24 h later, the cells were transfected with Myc-IRF3 and Flag-TRIM26 or control vector for 24 h, cell lysate was prepared and western blot analysis was performed with indicated antibodies. Similar results were obtained from three independent experiments.
(TIF)
