# Supplementary figures and images for: TRIM26 Negatively Regulates Interferon-β Production and Antiviral Response through Polyubiquitination and Degradation of Nuclear IRF3
Source: PLoS Pathog. 2015 Mar 12;11(3):e1004726. doi: 10.1371/journal.ppat.1004726 (PMC4357427; doi:10.1371/journal.ppat.1004726)

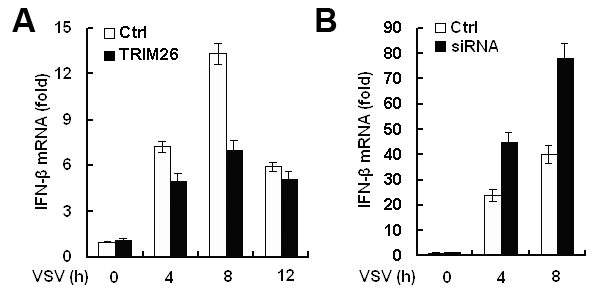

Supplement: S1 Fig — (A) Expression of IFN-β mRNA in Hela cells transfected with Flag-TRIM26 expression plasmid or control vector (Ctrl) followed with infection with VSV for indicated times. (B) Expression of IFN-β mRNA in mice peritoneal macrophages transfected with TRIM26 siRNA or control siRNA (Ctrl) followed with infection with VSV for indicated times. Data are representative of three independent experiments (mean ± S.D. of duplicates in A and B). (TIF) [file ppat.1004726.s001.tif]

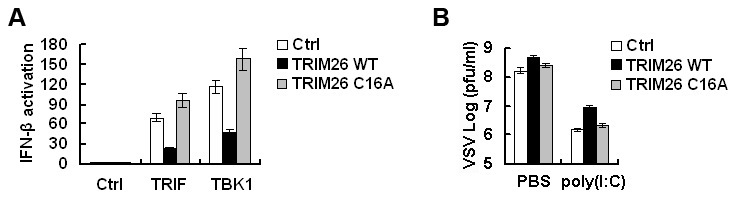

Supplement: S2 Fig — (A) HEK293 cells were transfected with expression plasmids for TRIF or TBK1, along with IFN-β reporter plasmid and TRIM26 WT or C16A plasmid, and analyzed luciferase activity. (B) HEK293 cells were transfected with expression plasmids for TRIM26 WT, C16A or control vector (Ctrl). 24 h later, cells were further transfected with poly(I:C) or left untreated. 18 h after poly(I:C) transfection, cells were infected with VSV (MOI, 0.1), and the supernatants were harvested at 12 h post-infection. Supernatants were analyzed for VSV titers with standard plaque assays. Data are representative of three independent experiments (mean ± S.D. of quadruplicates in A and triplicates in B). (TIF) [file ppat.1004726.s002.tif]

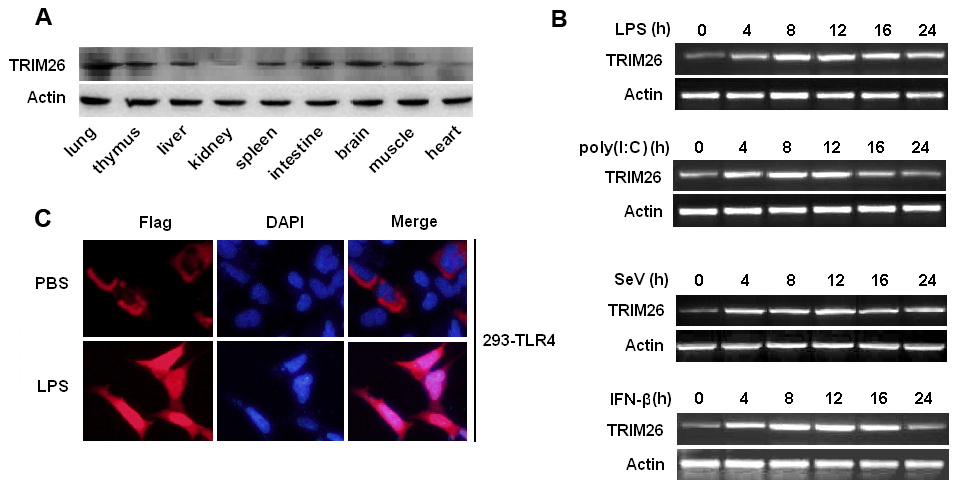

Supplement: S3 Fig — (A) Western blot analysis of TRIM26 protein expression in different mouse tissues. (B) RT-PCR analysis of TRIM26 mRNA expression in peritoneal macrophages stimulated with LPS, poly(I:C), IFN-β or infected with SeV for indicated times. (C) Immunofluorescent images of HEK293/TLR4 cells transfected with Flag-TRIM26 plasmid and then stimulated with LPS for 1 h. Flag-tagged TRIM26 was analyzed by immunostaining with anti-Flag antibody (red). Data are representative of three independent experiments. (TIF) [file ppat.1004726.s003.tif]

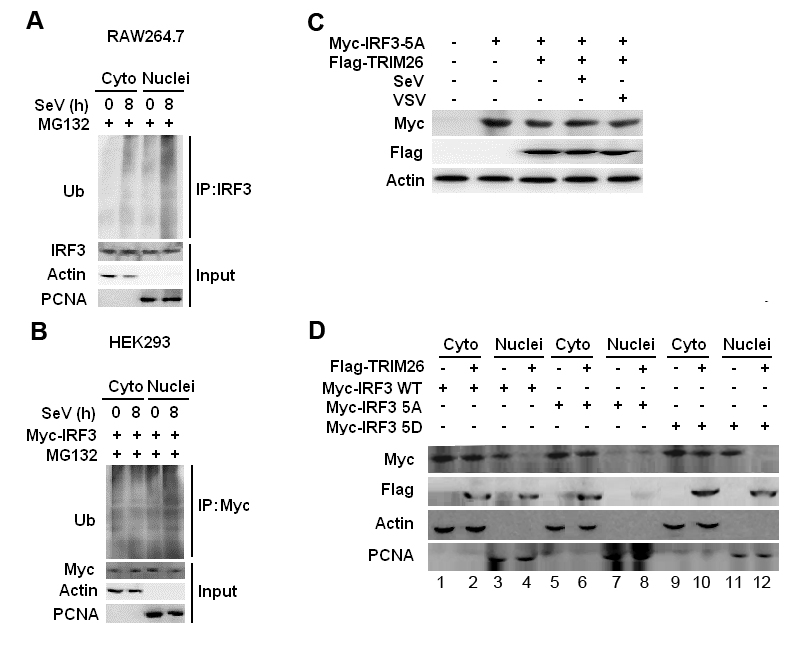

Supplement: S4 Fig — (A) Nuclear and cytoplasmic fractions prepared from RAW264.7 cells after infection with SeV were subjected to immunoprecipitation with anti-IRF3 antibody followed by western blot analysis with anti-Ub antibody. (B) Nuclear and cytoplasmic fractions from HEK293 cells transfected with Myc-IRF3 plasmid followed by SeV infection were subjected to immunoprecipitation with anti-Myc antibody followed by western blot analysis with anti-Ub antibody. (C) Western blot analysis of Myc-IRF3 5A expression in HEK293 cells transfected with expression plasmid for Myc-IRF3 5A and Flag-TRIM26 followed with VSV or SeV infection. (D) Western blot analysis of IRF3 expression in cytoplasmic and nuclear fractions prepared from HEK293 cells transfected with Myc-IRF3 WT, 5A, 5D together with Flag-TRIM26. Similar results were obtained from three independent experiments. (TIF) [file ppat.1004726.s004.tif]

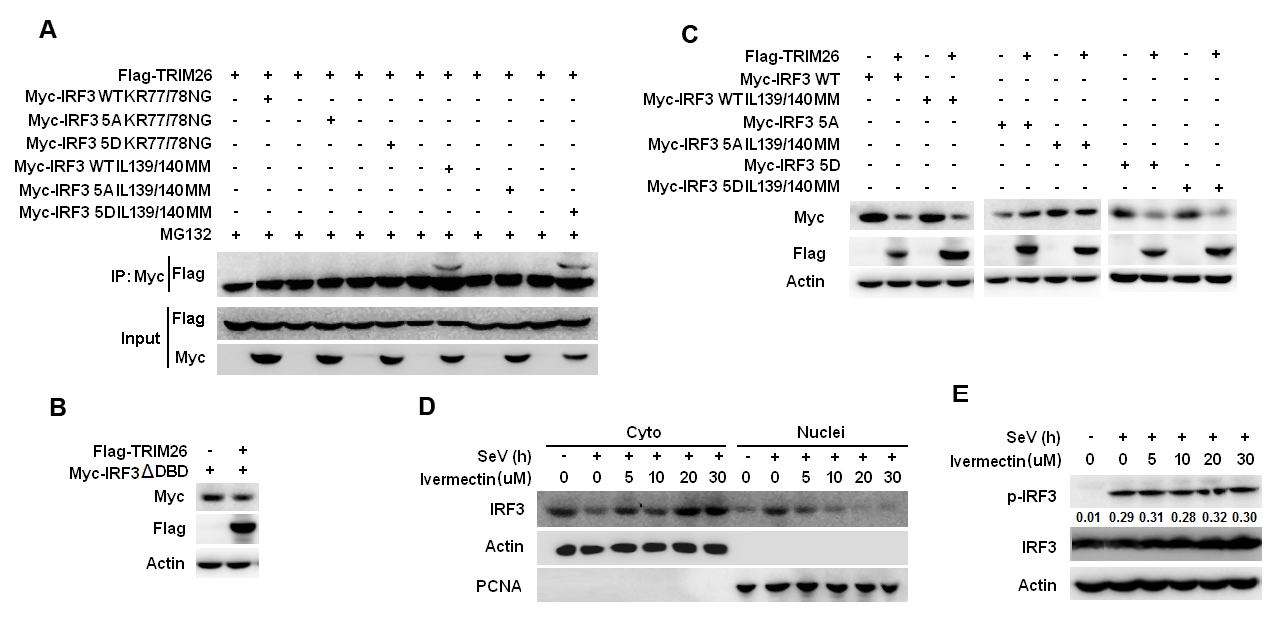

Supplement: S5 Fig — (A) Lysates from HEK293 cells transfected with Flag-TRIM26, IRF3 NLS mutants and NES mutants or control vector and followed with treatment with MG-132, were subjected to immunoprecipitation with anti-Myc antibody followed by western blot analysis with anti-Flag antibody. (B) Western blot analysis of the expression IRF3 mutant ΔDBD in HEK293 cells transfected with Myc-IRF3 ΔDBD and Flag-TRIM26 or control vector. (C) Western blot analysis of IRF3 expression in HEK293 cells transfected with Myc-IRF3 WT and various mutants in the NES. (D) Western blot analysis of IRF3 protein in cytoplasmic and nuclear fractions prepared from HEK293 cells pretreated with different concentrations of Ivermectin for 2 h, followed by SeV infection for 4 h. (E) Western blot analysis of phosphorylated IRF3 and total IRF3 protein in HEK293 cells pretreated with different concentrations of Ivermectin for 2 h, followed by SeV infection for 4 h. Similar results were obtained from three independent experiments. (TIF) [file ppat.1004726.s005.tif]

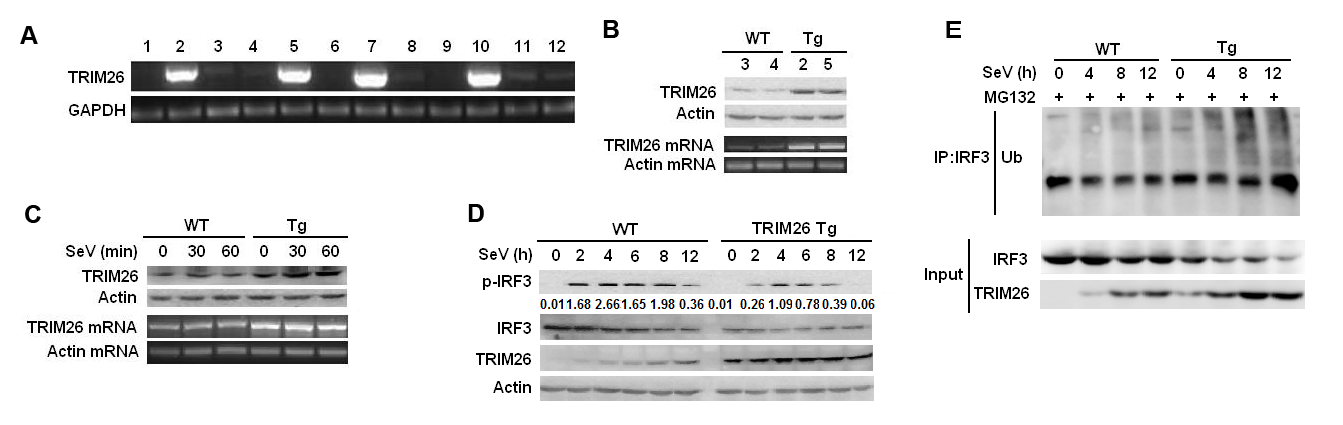

Supplement: S6 Fig — (A) TRIM26 transgenic mice were identified by PCR assays of genomic DNA from tails of mice. (B) RT-PCR and western blot analysis of the expression of TRIM26 mRNA and protein in the thymus from the TRIM26-Tg mice and WT mice identified in (A). (C) RT-PCR and western blot analysis of the expression of TRIM26 mRNA and protein in peritoneal macrophages from TRIM26-Tg mice and WT mice before and after SeV infection. (D) Western blot analysis of phosphorylated-IRF3, total IRF3 and TRIM26 in peritoneal macrophages from TRIM26-Tg and WT mice infected with SeV for indicated times. (E) Lysates prepared from peritoneal macrophages from TRIM26-Tg and WT mice pretreated with MG-132 for 2 h and then infected with SeV for indicated times, were subject to IP with IRF3 antibody followed by IB with Ubiquitin antibody. Data are representative of five (A) and three (B-E) independent experiments. (TIF) [file ppat.1004726.s006.tif]

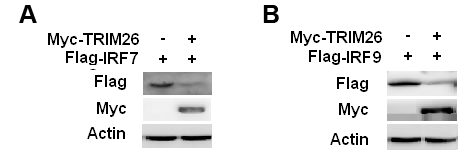

Supplement: S7 Fig — Western blot analysis of IRF7 and IRF9 expression in HEK293 cells transfected with Flag-IRF7 (A) and Flag-IRF9 (B) together with Myc-TRIM26 or control vector. Similar results were obtained from three independent experiments. (TIF) [file ppat.1004726.s007.tif]

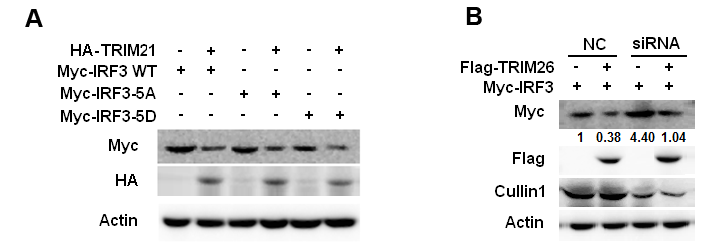

Supplement: S8 Fig — (A) Western blot analysis of Myc-IRF3 expression in HEK293 cells transfected with expression plasmid for Myc-IRF3 WT, 5D and 5A together with HA-TRIM21 or control vector. (B) Cullin 1 specific siRNA or control siRNA were transfected into HEK293 cells, 24 h later, the cells were transfected with Myc-IRF3 and Flag-TRIM26 or control vector for 24 h, cell lysate was prepared and western blot analysis was performed with indicated antibodies. Similar results were obtained from three independent experiments. (TIF) [file ppat.1004726.s008.tif]
